# Profile of Acute Infectious Markers in Sporadic Hepatitis E

**DOI:** 10.1371/journal.pone.0013560

**Published:** 2010-10-21

**Authors:** Shoujie Huang, Xuefeng Zhang, Hanmin Jiang, Qiang Yan, Xing Ai, Yijun Wang, Jiaping Cai, Lang Jiang, Ting Wu, Zhongze Wang, Li Guan, J. Wai Kuo Shih, Mun-Hon Ng, Fengcai Zhu, Jun Zhang, Ningshao Xia

**Affiliations:** 1 National Institute of Diagnostics and Vaccine Development in Infectious Diseases, Xiamen University, Xiamen, China; 2 Department of Preventive Medicine, Medical College of Xiamen University, Xiamen, China; 3 Jiangsu Provincial Center for Disease Control and Prevention, Nanjing, China; 4 Dongtai Center for Disease Control and Prevention, Dongtai, China; BMSI-A*STAR, Singapore

## Abstract

Laboratory diagnosis of acute infection of hepatitis E virus (HEV) is commonly based on the detection of HEV RNA, IgM and/or rising IgG levels. However, the profile of these markers when the patients present have not been well determined. To clarify the extent of misdiagnosed sporadic hepatitis E in the initial laboratory detection, serial sera of 271 sporadic acute hepatitis cases were collected, detected and the dynamics of each acute marker during the illness course were analyzed. 91 confirmed cases of hepatitis E were identified based on the presentation of HEV RNA, IgM or at least 4 fold rising of IgG levels. 21 (23.1%) hepatitis E cases were false negative for the viral RNA and 40 (44.0%) for rising IgG, because occurrence of these markers were confined to acute phase of infection and viremia had already subsided and antibody level peaked when these patients presented. IgM was detected in 82 (90.1%) cases. It is the most prevalent of the three markers, because the antibody persisted until early convalescence. Nine cases negative for IgM were positive for rising IgG and one was also positive for the viral RNA; all of these nine cases showed high avid IgG in their acute phase sera, which indicated re-infection. In summary, it is not practicable to determine the true occurrence of sporadic hepatitis E. Nevertheless, it could be closely approximated by approach using a combination of all three acute markers.

## Introduction

Hepatitis E Virus (HEV) has been recognized to be a major cause of outbreaks associated with fecal contamination of drinking water for decades [1], [2], [3], [4], [5], [6], [7]. As better diagnostic assays become commercially available, this pathogen is now recognized also as a major etiologic agent of sporadic acute hepatitis in endemic countries and autochthonous acute hepatitis cases in Western Europe and industrialized countries of East Asia [1], [8]. Hepatitis E appears to be rare in the United States, despite the finding of relatively high seroprevalence in various populations [9], [10], [11], [12]. The reason is not well understood, but it is at least partly because of a lack of a FDA-licensed diagnostic assay.

The virus afflicting humans consists of a single serotype and 4 major genotypes. Genotypes 1 and 2 have only been isolated from humans and are mainly distributed in developing countries. In this setting they cause large water borne outbreaks and sporadic cases and are associated with a high mortality among pregnant women and individuals with chronic liver disease [13], [14], [15]. Genotypes 3 and 4 are zoonotic with swine being the principal reservoir. The virus is widely distributed, causing limited food-borne outbreaks and sporadic cases, affecting mainly middle aged and elderly males [1], [16], [17].

Hepatitis E is diagnosed by detecting viral RNA (RT-PCR) in the serum and/or feces during the incubation period or early acute phase of disease, or, more commonly, by demonstrating IgM anti-HEV or a rising titer of IgG anti-HEV in the serum during the late acute phase or convalescent phase of the illness [8]. While generally considered to be specific, the sensitivity of these markers has not been determined. Consequently, the proportion of hepatitis E cases that has missed diagnosis is uncertain. To clarify the extent of misdiagnosed sporadic hepatitis E in the initial laboratory detection, serial sera of 271 sporadic acute hepatitis cases were collected, detected and the dynamics of acute markers during the illness course were analyzed.

## Results

### Diagnosis and Exclusion of Hepatitis E

**Figure 1:**
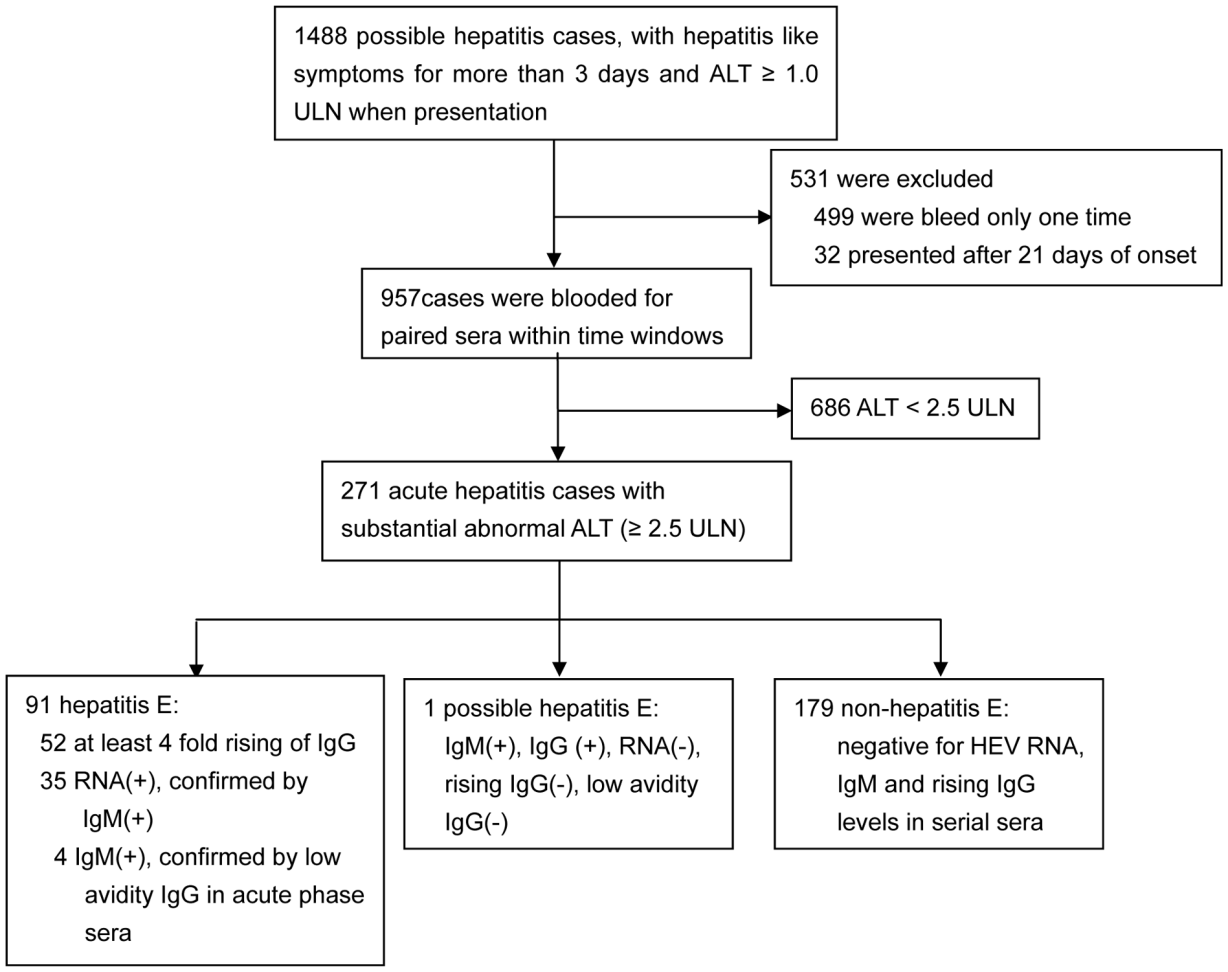
Flowchart of acute hepatitis patients diagnosed. Among 1488 patients presenting with fatigue and loss of appetite for no less than 3 days, 91 were diagnosed as hepatitis E, with a positive finding for at least one of the three HEV acute markers. Noted that false negative for any one of the acute viral markers was compensated for by a positive finding for one or both or the other markers.

**Figure 2:**
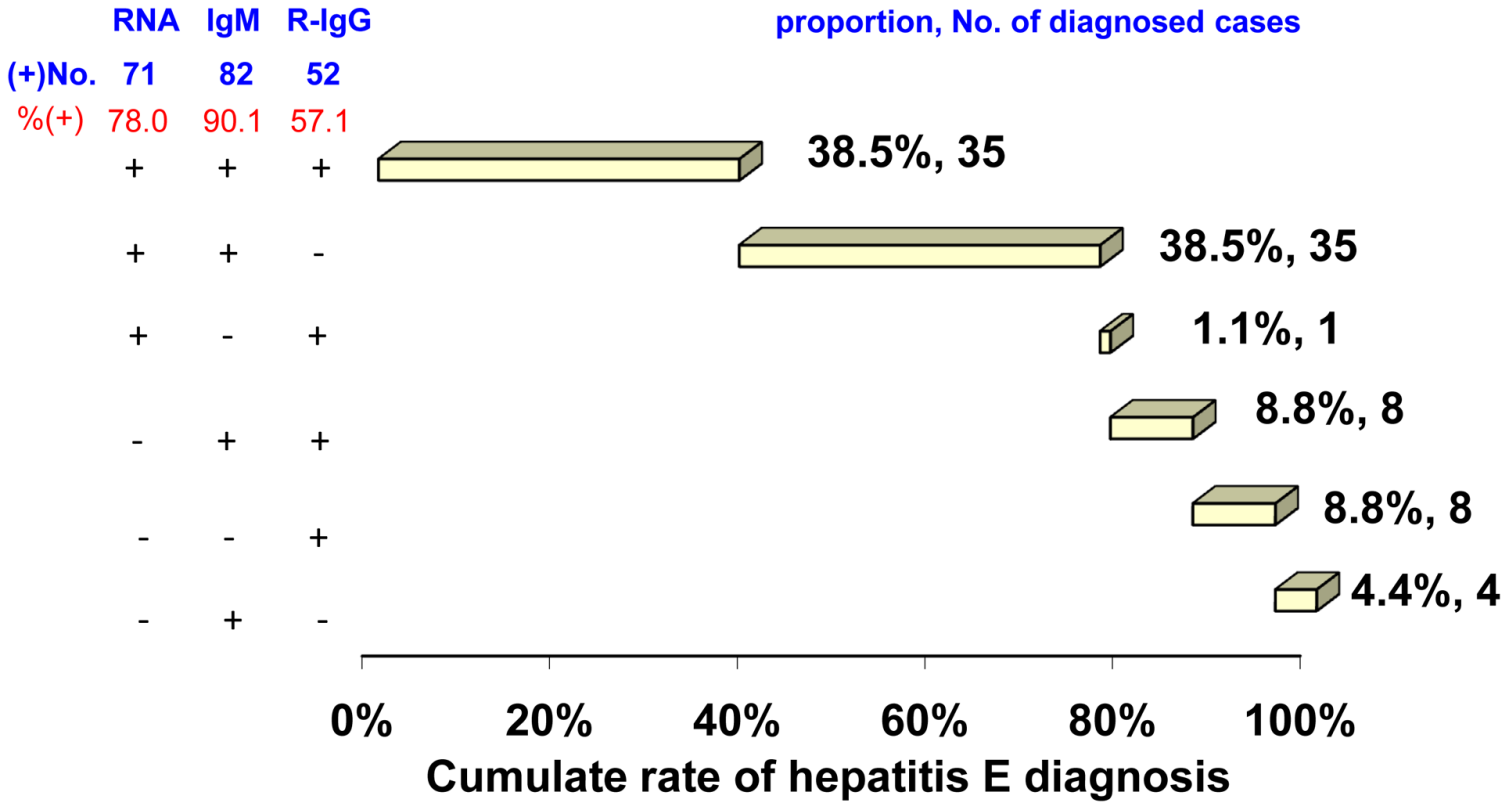
Distribution of acute markers among hepatitis E patients. Among 271 patients presenting with fatigue and loss of appetite attended by elevated serum ALT levels, 91 were diagnosed as having hepatitis E, with a positive finding for at least one of the three HEV acute markers. Noted that false negative for any one of the acute viral markers was compensated for by a positive finding for one or both or the other markers.

1488 sporadic possible hepatitis cases presenting with complaining of fatigue and/or loss of appetite for at least 3 days were enrolled (Figure 1). Serial sera were collected and detected for HEV RNA, IgM and IgG levels from 271 acute hepatitis cases whose liver injury were evidenced on presentation by ALT levels ≥2.5 ULN. 91 cases of hepatitis E were confirmed based on the presentation of at least 4 fold rising of IgG levels, RNA, IgM or low avidity IgG (Figure 1 and Figure 2). They include 3 who were co-infected with HBV, being also positive for HBc IgM.

Among 91 hepatitis E cases, acute marker profiles of 82 cases are compatible with primary infection, reflecting a vigorous IgM response, a relatively weak and transient IgG response with production of low avidity IgG and a relatively protracted viremia. The remaining 9 cases were positive for rising IgG levels and one was also positive for RNA, but all were negative for both IgM and low avidity IgG (Table 1). Such restricted profiles are compatible with re-infection [18], [19]. Of the 71 viral RNA positive cases, 70 underwent sequencing and 66 (94.3%) were genotype 4, the remaining 4 isolates were genotype 1.

**Table 1:** Serological profiles of hepatitis E patients negative for IgM anti-HEV.

Case code	Days after onset	ALT (ULN)	HEV RNA	IgM (S/Co)	IgG (Wu/ml)	IgG avidity level (%)*
GY0630	10	28.7	−	0.0	0.5	ND
	116	0.7	−	0.1	16.0	84.5
GY1311	8	2.6	−	0.0	0.6	79.2
	24	2.3	−	0.4	6.0	84.9
GY1347	10	6.0	−	0.1	3.0	74.8
	24	1.2	−	0.1	15.7	73.7
GY2835	3	7.0	−	0.1	<0.03	N/A
	24	0.8	−	1.0	0.7	82.8
GY3208	6	8.6	−	0.1	<0.03	N/A
	70	5.6	−	0.7	2.8	51.9
GY4209	4	6.0	−	0.0	0.2	107.5
	29	1.2	−	0.1	1.0	70.0
GY5556	6	2.8	−	0.1	<0.03	N/A
	22	0.9	−	0.1	0.7	74.0
GY5761	5	5.5	−	0.0	1.5	81.0
	39	0.5	−	0.7	15.7	65.2
GY3083	6	7.0	+	0.2	<0.03	N/A
	38	1.5	−	0.5	8.1	80.7%

*Low avidity antibody was indicated, when the residual antibody levels determined in the presence of 5 M urea was ≤50% of the corresponding control levels concurrently determined in the absence of urea. ND, not detected. N/A, not applicable.

One patient had marginal level of IgM anti-HEV on day 3 (with IgM S/Co of 2.1) and day 26 (with IgM S/Co of 1.7), but negative for HEV RNA. The IgG anti-HEV was at low level, had no notable rising (3.8 Wu/ml on day 3 and 3.7 Wu/ml on day 26), and the avidity were high (72.6% on day 3 and 79.0% on day 26). Hence this case was defined as possible hepatitis E case.

The remaining 179 cases were negative for all three markers simultaneously, hence they were diagnosed as non-E hepatitis, included 2 cases positive for HAV IgM, 19 cases positive for HBc IgM and 3 cases positive for HCV IgG.

### Dynamics of acute markers during progression of illness

Table 2 summarized the prevalence of HEV RNA, IgM and IgG in the paired samples from hepatitis E cases. When presentation, HEV RNA, IgM and IgG were detectable in 68 (73.9%), 73 (79.4%) and 87 (94.6%) patients respectively. HEV RNA in most patients (89.7%, 61/68) converted to negative in the second samples. Because levels of HEV IgM already peaked when patients presented and the antibody persisted for longer period than the other markers, it is most commonly detected among hepatitis E patients and prevalence of the marker in the acute samples (79.4%) is similar to that in the convalescent samples (87.0%). The IgG anti-HEV can be detected in all hepatitis E patients, most (94.6%, 87/92) had reached high levels when their first presentation (Table 2).

**Table 2:** The prevalence of HEV RNA, IgM anti-HEV and IgG anti-HEV in the paired sera of hepatitis E patients.

Present of markers (n = 91)				
1^st^ sample	2^nd^ sample	RNA (%)	IgM (%)	IgG (%)
+	+	7(7.7)	70(76.9)	86(94.5)
+	−	61(67.0)	2(2.2)£	0(0.0)
−	+	3(3.3)*	10(11.0)	5(5.5)§
−	−	20(22.0)	9(9.9)	0(0.0)

*All three HEV RNA conversion cases were companied with anti-HEV IgM positive conversion and IgG rising of ≥4 folds.

£ Both were positive with HEV RNA.

§ All negative for anti-HEV IgM in their first samples. One case was positive with HEV RNA in first sample, and another case converted to anti-HEV IgM positive on his second sample.

**Figure 3:**
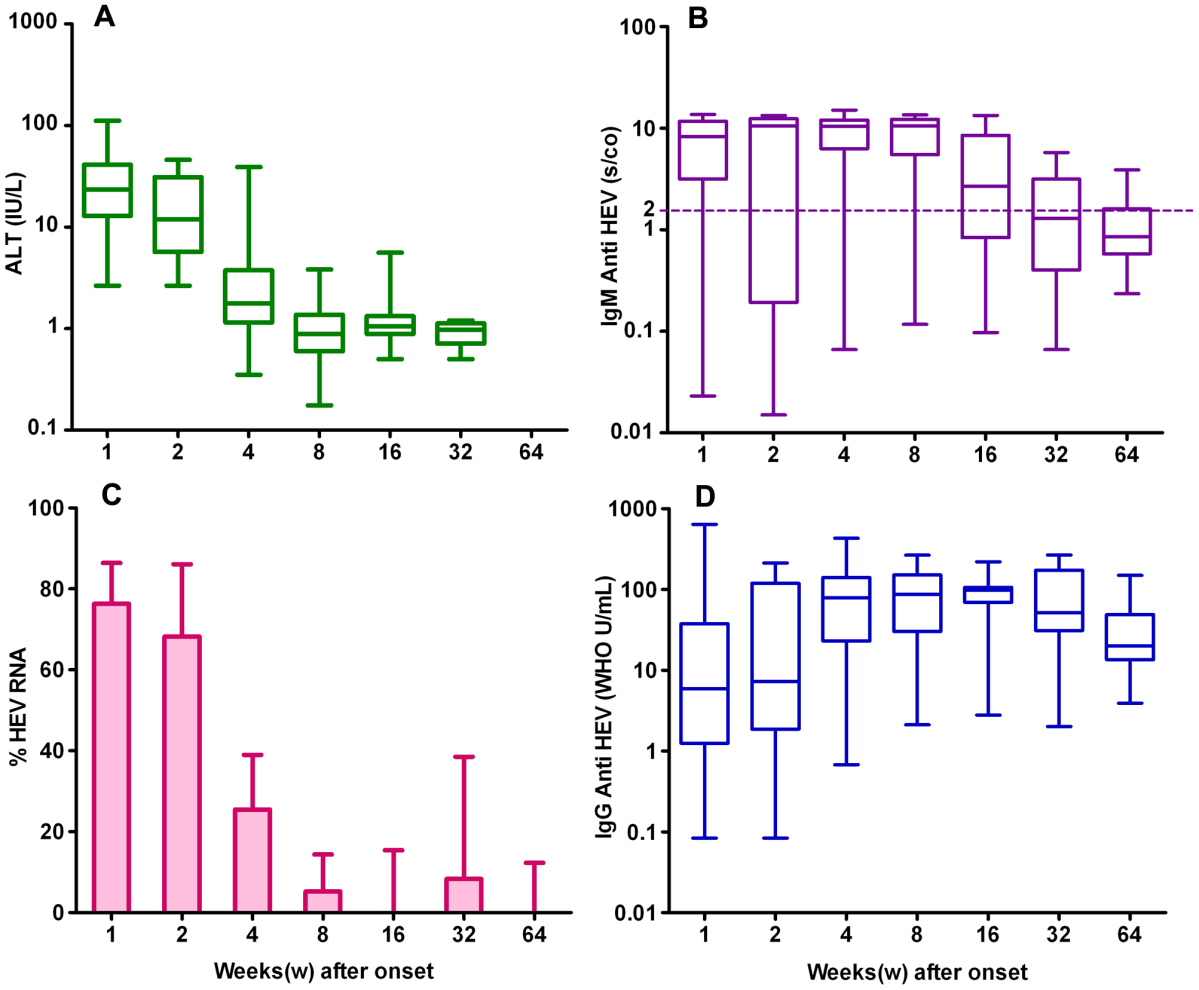
Dynamics of acute markers in acute hepatitis. Serum samples were collected from 91 consecutive hepatitis E cases within the first week of onset symptoms (n = 59) and in the subsequent intervals after onset of symptoms from: one to 2 weeks (n = 21); 2 to 4 weeks (n = 55); 4 to 8 weeks (n = 57); 8 to 16 weeks (n = 22); 16 to 32 weeks (n = 12) and 32 to 64 weeks (n = 28). The levels of ALT(A), IgM anti-HEV(B) and IgG anti-HEV(D) of the different groups of serum samples are shown as range (whiskers), interquartile (boxes) and median (line within the boxes) values. The occurrence of the viral RNA(C) was shown as % prevalence (bar) and 95% confidence interval (whiskers). Dotted line represents the cutoff levels of IgM anti-HEV (2.0 S/co).

Figure 3 shows ALT levels, prevalence of RNA and levels of IgM and IgG in serial sera taken from hepatitis E cases. It was evident that ALT levels were declining, viremia was subsiding and antibody response was already initiated, when these patients presented. Most patients achieved normalization of ALT levels and virus clearance within 4 weeks of onset of symptoms. HEV IgM levels already peaked when patients presented and remained at high levels for 8 weeks. Level of the antibody declined rapidly thereafter, falling below the cut off level (dotted line) among most patients after 32 weeks. HEV IgG levels were rising when patients presented. The antibody reached peak levels about 4 weeks after onset of symptoms and maintained at high levels for more than one year. The mean peak level of IgG in hepatitis E cases was 94.4±2.4 Wu/ml. As contrast, among 118 non-hepatitis E patients who were positive with IgG anti-HEV in the initial sample, the geometry mean concentration was 1.2±4.4 Wu/ml.

### Performance of HEV Acute Markers

Diagnostic performance by rising IgG, RNA and IgM individually and in combination was assessed (Table 3). For calculation, the possible hepatitis E case was seemed as non-E case. Sensitivity of rising IgG was 57.1%. The mean presentation time of cases who showed rising IgG was 6.3±3.7 days post-onset, significantly earlier than that in cases negative for rising IgG, which was 8.0±4.1 days (p<0.05). It indicated that false negative of rising IgG associate with the delayed presentation. Sensitivity of RNA was 78.0%. However, the false negative of RNA cannot be contributed to the delay of presentation, because the RNA negative patients first presented at 6.6±3.3 days, similar as that of the RNA positive patients, 7.2±4.1 days (p>0.05). IgM afforded the highest sensitivity (90.1%) of the three markers; false negative was attributed to re-infection (Table 1). False negative for one marker was compensated for by a positive finding for one or both of the other markers, such that the sensitivity achieved by combination of two markers was higher than that achieved by either marker alone. The most complementary combination was IgM and rising IgG, as the former covers all cases attributable to primary infection and the latter covers all the cases attributable to re-infection. Detection of either rising IgG or RNA is considered diagnostic with specificity of 100%; the specificity of IgM under the present clinical setting was found to be 99.4%.

**Table 3:** Sensitivity, specificity, positive and negative predictive value of HEV acute markers in diagnosis of acute hepatitis patients.

Markers	Sensitivity (n = 91)		Specificity (n = 180)			
	(+) No.	Sensitivity (%) (95%CI)	(+) No.	Specificity (%) (95%CI)	Positive predictive value (%)(95%CI)	Negative predictive value (%)(95%CI)
IgM	82	90.1(82.1–95.4)	1	99.4(96.9–100.0)	98.9(93.5–100.0)	95.2(91.1–97.8)
RNA	71	78.0(68.1–86.0)	0	100.0(98.0–100.0)	100.0(94.9–100.0)	90.0(85.0–93.8)
Rising IgG*	52	57.1(46.3–67.5)	0	100.0(98.0–100.0)	100.0(93.2–100.0)	82.2(76.5–87.0)
IgM or RNA	83	91.2(83.4–96.1)	1	99.4(96.9–100.0)	98.8(93.5–100.0)	95.7(91.8–98.1)
IgM or rising IgG	91	100(96.0–100.0)	1	99.4(96.9–100.0)	98.9(94.1–100.0)	100.0(98.0–100.0)
RNA or rising IgG	87	95.6(89.1–98.8)	0	100.0(98.0–100.0)	100.0(95.9–100.0)	97.8(94.5–99.4)
IgM, RNA, or rising IgG	91	100 (N/A) &	1	99.4(96.9–100.0)	98.9(94.1–100.0)	100 (N/A) &

*IgG anti-HEV seroconversion or showed a ≥4 fold rising of IgG anti-HEV.

& The sensitivity was set to be 100%, so the calculation for 95% CI is not applicable (N/A).

## Discussion

HEV RNA is most commonly used as acute markers in routine diagnosis of hepatitis E. IgM anti-HEV was increasingly used in routine diagnosis since its reliability been dramatically increased by using antigens that contained immono-dominant epitopes of the virus and became commercially available in many countries recently [18], [19], [20], [21], [22], [23], [24], [25]. Rising IgG is less frequently used, because it requires paired acute and convalescence serum samples and provides a retrospective diagnosis. Low avidity IgG was used to supplement findings by these three markers in some of the cases, but it was not used as a primary diagnostic marker in the present study, because its specificity has not yet been established.

However, most published data for the performance of these tests were obtained based on samples confirmed by viral RNA, or samples positive for at least one referenced assay [19], [21], [22], [23], [24], [25]. Hence the false negative rates of RNA tests cannot be evaluated due to the sample selection strategies. In the mean time, bias was unavoidable when sensitivity and specificity be evaluated, because only samples from RNA detectable patients were selected. As we are aware, the present is the first detailed study to assess the performance of viral RNA, IgM and IgG anti-HEV markers in laboratory diagnosis of sporadic hepatitis E. In this study, paired acute and convalescent sera were obtained from acute hepatitis cases.

To avoid sampling bias, study subjects were consisted of 271 cases of acute hepatitis consecutively presented over 12 months period with complaints of constitutional signs, such as anorexia, fatigue and etc., for 3 days and abnormal ALT levels ≥2.5 ULN. The antigenic cross-reaction of human HEV with other pathogens had not been reported, hence substantially rise of IgG anti-HEV levels was generally considered as specific marker of acute infection. However, false positive of RNA or IgM might occur during operation. Therefore, diagnosis of hepatitis E based on RNA or IgM was further confirmed by each other or by the presence of low avidity IgG in acute phase serum. False positive diagnosis by this approach is considered unlike. Exclusion of hepatitis E was indicated by a negative finding for all the 3 markers simultaneously. The fecal samples of the patients had not been collected in this study. However, it is not likely that the sensitivity of RNA detection would be higher if fecal samples be detected, because the viral shedding period in the serum is usually similar to or a little longer than that in the feces in most hepatitis E patients [26].

91 patients were diagnosed with hepatitis E. Occurrence of rising IgG and viremia was largely confined to the acute phase of the infection during the first 4 weeks of onset of symptoms. Onset of symptoms is innocuous, however, and presentation was delayed to up to 21 days after onset of symptoms, when IgG antibody levels had already peaked among 42.9% of patients and viremia had subsided among 22.0% of patients. HEV IgM persisted for the longest period until early convalescence about 16 weeks after onset of symptoms and the marker was detected among 82 of the patients. Detection of this marker was variously accompanied by at least one of the other two markers or by the presence of low avidity IgG antibody, reflecting a vigorous IgM response attended by production of low avidity IgG which is compatible with primary infection. The remaining 9 patients yielded a positive finding for rising IgG and one was also positive for viremia, but all were negative for both IgM and low avidity IgG. These acute marker profiles reflect a weak IgM response attended by production of avid IgG compatible with re-infection.

Under the above described clinical setting, it was evident that diagnosis is best achieved by combination of all 3 markers. Sensitivity achieved by this approach depends on clinical settings; it is highly sensitive (100.0%) for cases attributed to primary infection, but substantially lower (57.1%) for cases attributable to re-infection, supposed due to the natural appearance of re-infection as limited viral replication and limited induction of IgM anti-HEV. In present study area, which is endemic for HEV genotype 4, assume the sensitivity of rising IgG for re-infection is similar as that for primary infection (i.e., 57.1%), and the sensitivity of RNA for re-infection remains to be 11.1% (1/9), then the combined sensitivity of RNA and rising IgG is 61.9%, the expected number of re-infection is 14.5. Under this setting, the expected hepatitis E among 271 acute hepatitis were 96.5 instead of 91; the overall sensitivities of three acute markers were estimated to be 73.6%, 85.0% and 53.9% for RNA, IgM and rising IgG respectively. The sensitivity of combination of the three acute markers is estimated to be 94.3% and negative predictive value is 96.8%; failure of diagnosis occurred mainly in re-infection.

The approach in the present study affords a retrospective diagnosis and is valuable for epidemiologic studies. In routine practice, diagnosis is commonly based on detection of HEV RNA and/or IgM usually in single acute samples. False negative associated with such routine approach is mainly due to re-infection and sensitivity achieved by combination of both markers was estimated to be 86.0%, which is very similar as that of 85.0% achieved by HEV IgM alone. Sensitivity of HEV RNA and rising IgG is substantially lower (73.6% and 53.9% respectively), because viremia has already subsided and/or antibody levels had already peaked in substantial number of patients at presentation. In low endemic areas such as in Europe and the US, on the other hand, sensitivity achieved by these markers is expected to be higher, because re-infection would be less common.

In conclusion, the results show that it is not practicable to determine the true occurrence of sporadic hepatitis E. Nevertheless, it could be closely approximated by approach using combination of all three acute markers. Deviation from true occurrence of hepatitis E is higher for high endemic than low endemic areas.

## Materials and Methods

### Patients

In a group of community clinics and hospitals in rural townships in eastern China, 1488 possible hepatitis patients who suffered from fatigue and/or loss of appetite for at least 3 days and had an elevated alanine-leucine transaminase (ALT) level were enrolled. Informed consent in writing was obtained from each patient and the study protocol conformed to the ethical guidelines of the 1975 Declaration of Helsinki. Independent Ethics Committee approval was obtained from the Ethics Committee of Jiangsu Provincial Center for Disease Control and Prevention.

Acute hepatitis was defined as an acute liver damage evidenced on presentation by abnormal ALT levels ≥2.5 fold upper limit of the normal levels (ULN). Acute hepatitis cases whose presented sera were collected during time window of 3 to 21 days post-onset and were collected for at least one another follow-up sera were included for final analysis. Serial sera were collected from 271 of such consecutive cases at 3 to 21 days after onset of symptoms (6.7±3.7 days), and during convalescence 9 to 136 days after onset of symptoms (25.6±18.1 days). Additional serum samples were collected from some of the patients (n = 87) at later times up to one year after onset of symptoms.

Confirmed hepatitis E cases were defined as: 1) at least 4 fold rising IgG levels in paired sera; 2) RNA(+), confirmed by IgM(+); or 3) IgM(+), confirmed by low avidity IgG in acute phase serum. A possible hepatitis E case was defined as positive for HEV RNA or IgM but can not be confirmed or excluded by antibody dynamics.

### Detection of HEV RNA

Serum samples were tested for the presence of the HEV RNA by reverse-transcript PCR (RT-PCR) as previously described [27]. Briefly, total RNA was extracted from 200 µl of sample with Trizol (Invitrogen). A 150-nt segment of open-reading frame 2 (ORF2), was amplified using primers E1 (5′-CTGTTTAA(C/T) CTTGCTGACAC-3′, nt6260–6279) and E5 (5′-(A/T)GA(A/G) AGCCAAAGCACATC-3′, nt6568–6551) in the first round of PCR and primers E2 (5′-GACAGAATTGATTTCGTCG-3′, nt6298–6316) and E4 (5′-TG(C/T)TGGTT (A/G)TC(A/G)TAATCCTG-3, nt6486–6467) in the second round. PCR cycling conditions for both rounds consisted of 35 cycles of denaturation at 94°C for 30 sec, annealing at 53°C for 30 sec, and extension at 72°C for 40 sec.

### Detection of antibodies against HEV

The levels of IgM or IgG antibody against HEV were determined by commercial enzyme-linked immunosorbant assays (ELISA) (Beijing Wantai Biological Pharmacy Enterprise Co., Ltd., Beijing, China) according to the manufacturer's instructions. These kits are based on a recombinant antigen contained conformational immuno-dominant epitopes corresponding to amino acid residues 394 to 606 of the major structural protein specified by ORF 2 of the HEV genome [18], [20], [28], [29]. IgG anti HEV was expressed in world health organization unit (Wu) by comparing with the level of an assay reference serum of 16.5 Wu/ml and the limit of detection by the assay was 0.03 Wu/ml [30]. IgM anti-HEV was expressed as ratio to the cut-off value recommended by the manufacturer (S/Co) with a positive being S/Co ≥2. IgG anti-HEV avidity test was conducted on some serum samples as previously described [18], [31]. Briefly, serum samples were titrated in parallel in the presence and absence of 5 M urea. Avidity of IgG anti-HEV was expressed as percent of residual antibody levels determined in the presence of 5 M urea relative to the corresponding levels determined in parallel in the absence of 5 M urea. Presence of low avidity HEV IgG is indicated, when residual antibody levels were ≤50% of control values.

All specimens were also tested for IgM antibody against hepatitis A virus (HAV-IgM), IgM antibody against hepatitis B core protein (HBc-IgM), hepatitis B virus surface antigen (HBsAg), and antibody against hepatitis C virus (HCV-Ab) using commercial ELISA kits (Beijing Wantai Biological Pharmacy Enterprise Co., Ltd., Beijing, China).

### Statistical analysis

Data were expressed as mean ± SD. Student's t test was used for continuous variables. Differences were considered to be significant for P value <.05. The statistical analysis was performed using OpenEpi (Open Source Epidemiologic Statistics for Public Health, Version 2.3. www.OpenEpi.com, updated 2009/20/05, accessed 2009/01/10).
